# Cyclic hydrostatic pressure and cotton particles stimulate synthesis by human lung macrophages of cytokines in vitro

**DOI:** 10.1186/1465-9921-10-44

**Published:** 2009-06-02

**Authors:** Sarah Lewis, Dave Singh, Carol E Evans

**Affiliations:** 1 Tissue Injury and Repair Group, School of Clinical and Laboratory Sciences, Faculty of Medical and Human Sciences, University of Manchester, Stopford Building , Oxford Road, Manchester M13 9PT, UK; 2 NIHR Translational Research Facility, University Of Manchester, University Hospital Of South Manchester Foundation Trust, UK

## Abstract

**Background:**

Inhalation of particulates is a leading cause of the development of lung diseases and current understanding of the complex relationship between lung metabolism and airborne particulates is incomplete. It is well established that mechanical load is important in the development of the lung and in lung cell differentiation. The interaction between particle exposure and physical forces on alveolar macrophages is a physiologically relevant issue, but as yet understudied. This study examines the effect of cyclic hydrostatic pressure and cotton particles on synthesis of cytokines by human alveolar macrophages.

**Methods:**

Alveolar macrophages were obtained from patients with lung disease, either from lavage samples or from lung tissue resection. The commonly used cell line THP-1 was included in the experiments. Cell cultures were exposed to cotton particles and/cyclic hydrostatic pressure (3 or 5 psi); control cultures were exposed to medium only. TNFα, IL-1β and IL-6 were assayed in the culture media using specific ELISAs. Cells were characterized using morphology and markers specific for macrophages (Jenner/Giemsa staining, CD14 and CD68).

**Results:**

Exposure to cotton particles stimulated cytokine synthesis by macrophages from all three sources; exposure to cyclic hydrostatic pressure alone did not stimulate cytokine synthesis significantly. However, the combination of both particles and cyclic hydrostatic pressure increased the simulation of cytokine synthesis still further. Cell characterization demonstrated that the large majority of cells had a macrophage morphology and were positive for CD14 and CD68.

**Conclusion:**

These data suggest an interaction between cyclic hydrostatic pressure and particulate exposure, which increases alveolar macrophage cytokine production. This interaction was only observed at the higher cyclic hydrostatic pressure. However, in patient samples, there was considerable variation in the amount by which secretion of an individual cytokine increased and there was also variation in the mechanosensitivity of cells from the three different sources. Cyclic hydrostatic pressure, therefore, may be an important modulator of the response of alveolar macrophages to cotton particles, but the source of the cells may be a confounding factor which demands further investigation.

## Introduction

The lungs are continually subject to mechanical load, in the form of hydrostatic pressure and strain generated during inspiration and expiration. In this context, hydrostatic pressure is a load which deforms the tissue and cells by compression, whereas strain may be described as a load which causes elongation of the tissue and hence the cells within that tissue. The role of mechanical load in lung development [1,2] and lung cell differentiation [3] is now well established. However, although there have been several interesting studies on the effect of strain on lung cells [4-7], there have been few similar studies on the effect of load on lung cells [8]. Hydrostatic pressure may be elevated during increased ventilation, including forced ventilation, or pulmonary oedema. A recent publication by Garcia *et al *[9] described how physical forces affected the function and phenotype of cells in the lung. This review described the stimulation of cytokine synthesis by strain, by macrophages and lung epithelial cells and examines possible signalling pathways for such mechanotransduction.

Alveolar macrophages play a role in pulmonary inflammation in a variety of lung diseases. These cells are continually subject to mechanical load, but our knowledge of the response of these cells to such forces is sparse. We have previously shown macrophages from peripheral blood to be mechanoresponsive, causing a profound induction of the synthesis of proinflammatory mediators [10-14]. Furthermore, the pro-inflammatory effects of mechanical load forces on peripheral blood macrophages are enhanced by particulates.

Chronic environmental exposure to particulate matter can result in upregulation of the pro-inflammatory activity of alveolar macrophages. Examples of increased alveolar macrophage pro-inflammatory activity include chronic obstructive pulmonary disease (COPD) caused by cigarette smoking, and occupational cotton dust exposure which can cause byssinosis, chronic bronchitis or airflow obstruction. The interaction between particle exposure and physical forces on alveolar macrophages is a physiologically relevant issue, but as yet understudied.

The study reported here examined the effect of cyclic hydrostatic pressure (CHP) cotton particles or a combination of the two, on alveolar macrophages. We have evaluated the potential for CHP to modulate macrophage pro-inflammatory cytokine production, and the interaction between CHP and cotton particle exposure.

## Methods

### Patient samples

Five patients who were undergoing clinical investigational bronchoscopies were recruited, as well as 6 patients undergoing surgical resection for suspected or confirmed lung cancer. COPD was diagnosed based on a history of smoking for at least 10 pack years, typical symptoms (productive cough, breathlessness or wheeze), and airflow obstruction defined as FEV_1 _< 80% predicted, and FEV_1_/FVC ratio < 0.7. All subjects gave written informed consent. The study was approved by the local research ethics committee.

The 5 subjects undergoing bronchoscopy were all male and aged from 43–64 years. Three subjects were current smokers with normal lung function, while 2 were ex-smokers (1 with COPD).

The 6 subjects undergoing lung surgery were aged from 53 to 77 years; 5 male and one female. Four were current smokers (3 with COPD and 1 with normal lung function) and 2 were ex-smokers (both with normal lung function).

### Alveolar Macrophage Isolation

Broncho-alveolar lavage (BAL) was collected from the right lower lobe, or a lobe not affected by radiographic or endobronchial abnormalities: The bronchoscope was wedged in the right middle lobe and a maximum of 4 × 60 ml aliquots of prewarmed sterile 0.9% NaCl solution were instilled. The aspirated fluid was stored on ice before filtration (100 μm filter, Becton Dickenson). The filtrate was centrifuged (400 *g*/10 min at 4°C) and the cell pellet washed in RPMI 1640 medium supplemented with 2 mM L-glutamine, 100 U/ml penicillin, and 100 μg/ml streptomycin. BAL samples were collected and kept on ice to prevent cells sticking to the sample tube. Samples were filtered through a 100 μm cell sieve to remove debris then centrifuged at 1500 rpm (400 g) at 4°C for 10 minutes. The supernatant was discarded and cell count performed on the cell pellet.

Resected lung tissue was obtained from areas distant from the tumour, and perfused with 0.1 M NaCl to isolate macrophages. Lung tissue was perfused with 0.1 M Na Cl to isolate the cells before filtering and centrifuging as with the BAL samples. The supernatant was then discarded and cell counts performed on the cell pellet.

Macrophages were isolated from the mixed cell populations by the property of adherence. Cells were incubated in 20% Dulbecco's modified Eagles medium (DMEM, Invitrogen UK) + 1% Glutamine + 1% Penicillin/Streptomycin (Invitrogen UK) for 1 hour at 37°C in 5% CO_2_. Cell cultures were washed gently with phosphate-buffered saline (PBS) to remove any non-adherent cells. Approximately 80% of the white cells were found to be macrophages by this technique. The culture medium was replenished and the macrophages cultured for 24 hours before being exposed to experimental conditions.

### THP-1 Cell line

As this alveolar macrophage cell line is used extensively in research into the lung, we also performed loading experiments on THP-1 cells. The experimental protocol was the same as that used for the patient cells, except that, because of their increased sensitivity (vis-a-vis patient cells); THP-1 cells were seeded at a much lower density.

### Cotton Particulates

To examine the effect of typical cotton dust particles on these cells, Standard Cotton Dust used in all experiments. This is produced by the Cotton Incorporated company from crude cotton dust collected in a West Texas cotton mill between 1981 and 1983. This single source cotton dust allows the comparison of data and hypotheses from different scientific groups. The dust was analysed for endotoxin contamination using the Charles River Endosafe^® ^Portable Test System. This standard technique allows the quantitative detection of endotoxin by a kinetic chromagenic method, and involves the interaction of Limulus Amebocyte Lysate (LAL) and synthetic colour-producing substrate. This technique was performed at our laboratory under the guidance of a Charles River representative, using endotoxin free solutions and equipment.

**Figure 1:**
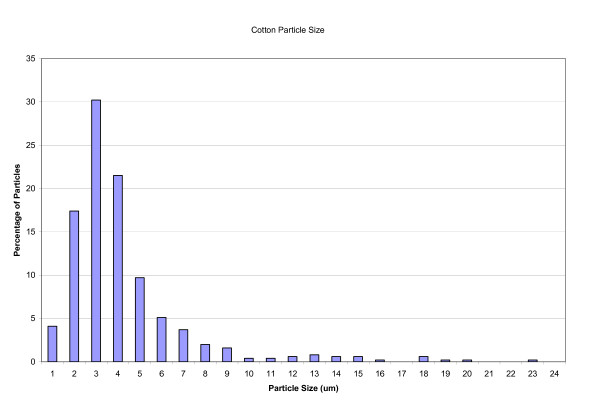
**A frequency diagram of size of cotton particles**.

Before being used in any experiments, 100 mg samples of cotton dust were sterilized by autoclaving and then suspended in 10 mls of the usual culture medium. This was filtered through a 40 μm cell sieve and the resulting filtrate, containing the smaller particles was used in the experiments. The size distribution of the filtered cotton particles was measured using image analysis and it was found that 22% of the measured particles had a diameter ≤2 μm and 94% ≤8 μm (Fig 1). The cotton particles used were of a size which has been shown previously to be the range phagocytosed by alveolar macrophages, evoking an inflammatory response [15,16].

### Cell characterization

The cells used in these studies were characterized using markers specific for macrophages. Cells were washed in PBS and fixed for 2 minutes in ice-cold ethanol (BDH, UK) prior to staining.

Histological staining, using the Jenner/Giemsa technique, was performed on bronchial lavage in order to establish the percentage of macrophages present in the samples. Briefly, lavage cell cytospins were immersed in Jenner solution (Raymond Lamb Ltd., UK, 0.3% in 100% methanol) for 2 minutes before immersing in Giemsa solution (Raymond Lamb ltd., UK, 1% in pH 6.4 buffer) for a further 20 minutes. Cytospins were then rinsed in pH 6.4 buffer, air-dried and mounted with Pertex. Leucocyte morphology and identification is clear using this technique, with macrophage nuclei staining purple and cytoplasm blue.

In addition, immunohistochemistry was performed using a commercially available antibody specific for CD68 (mouse anti-human CD68 diluted 1 in 100, Serotec Ltd. UK) and visualized using DAB (3,3 diamino bezidine, Sigma UK). CD68 is a glycoprotein found on the surface of macrophages, so cells staining positive for CD68 will therefore be macrophages.

### Cell culture and Pressurization

Macrophages from BAL or lung biopsies were seeded at 5 × 10^5^/ml into 24 well plates (1 ml/well) and incubated for 24 hours; THP-1 cells were seeded at 1 × 10^5^/ml. Culture media were then removed and 1 ml of fresh medium or 1 ml of the cotton dust/medium suspension was added to each well. The cultures were exposed to the cotton particles for 24 hours before pressurization and control cultures were exposed to medium only.

BAL, lung surgery macrophage and THP-1 cultures were loaded into our novel loading jig [10,11] and subjected to cyclic hydrostatic pressure (CHP). The pressure regime was a load of 3 psi, at a frequency of 2 seconds on/off for 1 hour. This load was in addition to atmospheric pressure psi [14.69]. Macrophages from the lung surgery samples and THP-1 cells were also exposed to 3 psi pressure (20.7 KPa) and/or cotton dust (<40 μm). Cultures were also exposed to a higher pressure of 5 psi (34.5 KPa) and/or cotton dust (<40 μm). The cultures were then returned to the incubator for 23 hours prior to analysis and the control (unloaded) cultures remained in the incubator throughout the experiment.

Culture media were removed from the cultures 23 hours post-pressure and cytokine levels were analysed by the ELISA technique. This was performed using commercially available ELISA kits from Diaclone (purchased from IDS Ltd Boldon, Tyne and Wear, UK). TNFα, IL-1β and IL-6 were assayed in the culture media from the patient's samples and TNFα was assayed in the culture media from the THP-1 cells.

As our previous research into CHP used peripheral blood macrophages, we compared the response of these cells and BAL macrophages to cotton dust exposure, collecting paired blood and lavage samples from our first few patients. However, it soon became apparent that peripheral blood macrophages underwent apoptosis on contact with the cotton dust particles, making any comparison impossible (data not shown).

### Statistical Analysis

Statistical analysis was performed on the results of both BAL macrophages and lung surgery macrophages using the non-parametric Friedman Test with Dunn Post Test. The parametric paired ANOVA with Bonferroni Post Test was performed on the results of the THP-1 experiments. Significance was defined as p < 0.05. Analyses were performed using Graphpad Instat 3.

## Results

### BAL macrophages

**Figure 2:**
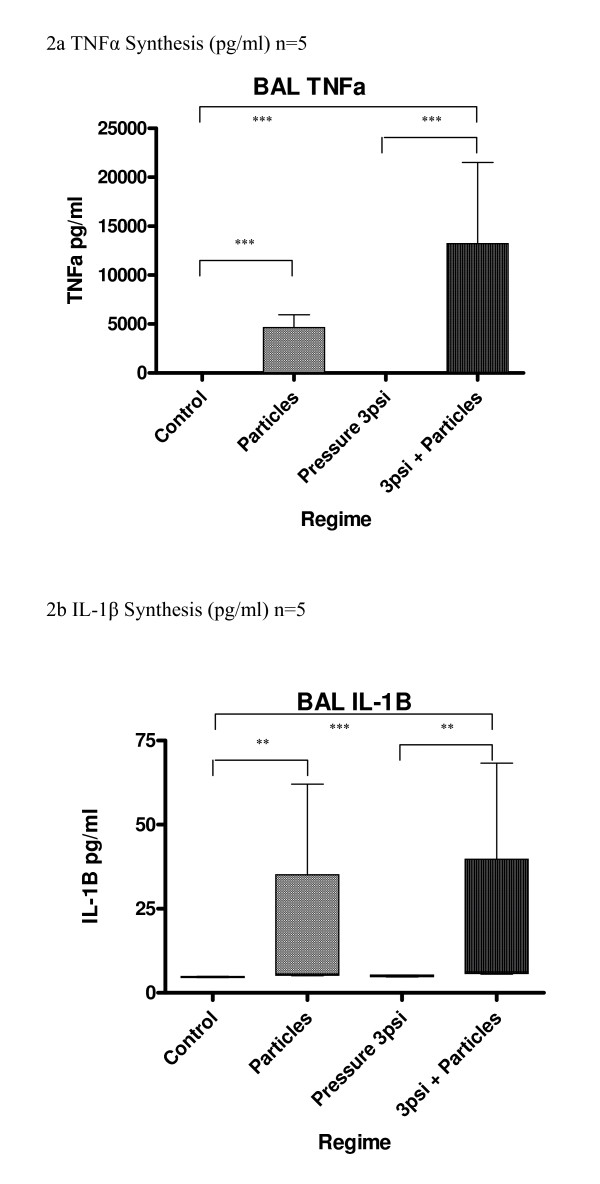
**(a-c) Effect of pressure and particles on synthesis of cytokines by BAL macrophages; data expressed as box and whisker plots with median line**. Comparison with unpressurized controls; ***p < 0.001, **p < 0.01, *p < 0.05

In cultures of BAL macrophages, synthesis of the cytokines TNFα, IL-1β and IL-6 was increased by exposure to cotton particles (Fig 2a–c). However, there was no significant increase in cytokine production caused by CHP alone at 3 psi. Cytokine levels after exposure to cotton particles and CHP were similar to particles alone.

### Lung Resection Macrophages

**Figure 3:**
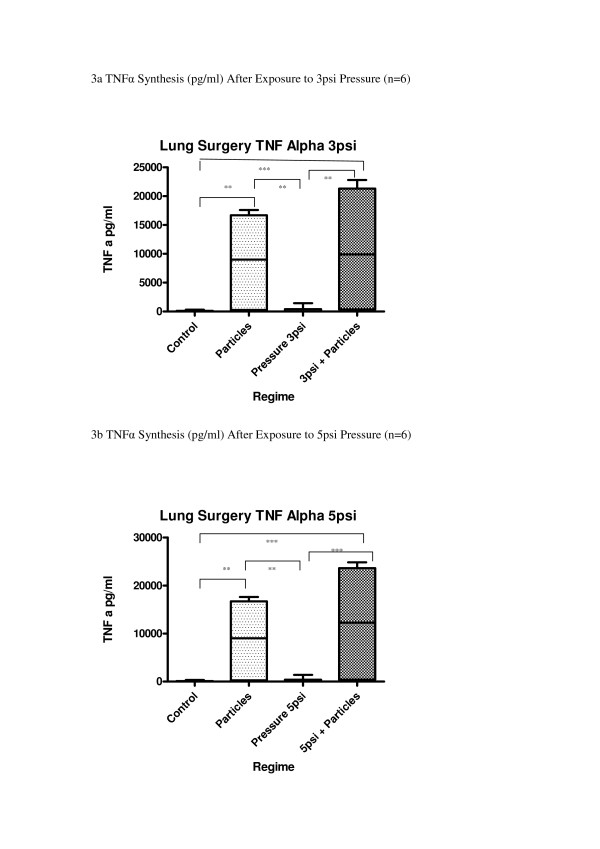
**(a-g) Effect of pressure and particles on synthesis of cytokines by Lung Surgery macrophages; data expressed as box and whisker plots with median line**. Comparison with unpressurized controls; ***p < 0.001, **p < 0.01, *p < 0.05

Lung resection alveolar macrophages released significantly greater levels of the cytokines TNFα, IL-1β and IL-6 after exposure to cotton particles (p < 0.001) (Fig 3a–f). CHP at either 3 psi or 5 psi had no statistically significant effect on the levels of these cytokines. (p > 0.05) although a trend towards increase in synthesis of cytokines was seen, when compared with unpressurized controls (TNFα 17%; IL-1β 6%; IL-6 26%).

Exposure to both pressure and cotton particles produced numerically the greatest response from the cultures, which reached statistical significance for IL-6 synthesis at 5 psi compared to pressure alone or particles alone (p < 0.01).

Comparison of the results from these two cell types showed that there was a significant difference in response between the two cell types when exposed to particles or both stimuli together (p < 0.01 in both cases) (Fig 3g). There was no significant difference between the two cell types when exposed to pressure alone; neither was there any difference to the control.

### THP-1 Cell line

**Figure 4:**
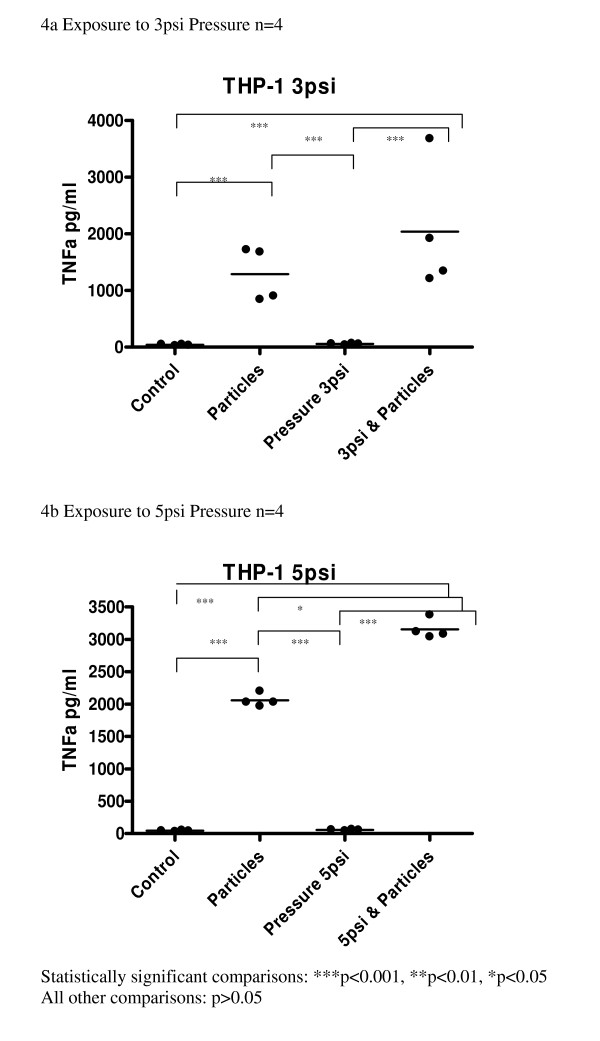
**(a-b) Effect of pressure and particles on synthesis of TNF-α by THP-1 macrophages; data expressed as scatter plots with mean line**. Comparison with unpressurized controls; ***p < 0.001, **p < 0.01, *p < 0.05

Cultures exposed to cotton particles showed a significant increase in synthesis of TNFα when compared to the controls (p < 0.001). Synthesis of TNFα was not increased when THP-1 cells were exposed to either level of CHP (3 psi or 5 psi) (Fig 4a, b).

Exposure to both CHP and cotton particles produced numerically the greatest response from the cultures. At 5 psi, TNFα production was significantly increased compared to particle exposure alone or pressure alone (p < 0.05).

### Cotton Particulates

Standard Cotton Dust was found to contain 17.37 EU/mg dust ≡ 1.74 ng endotoxin/ml dust.

### Cell characterization

**Figure 5:**
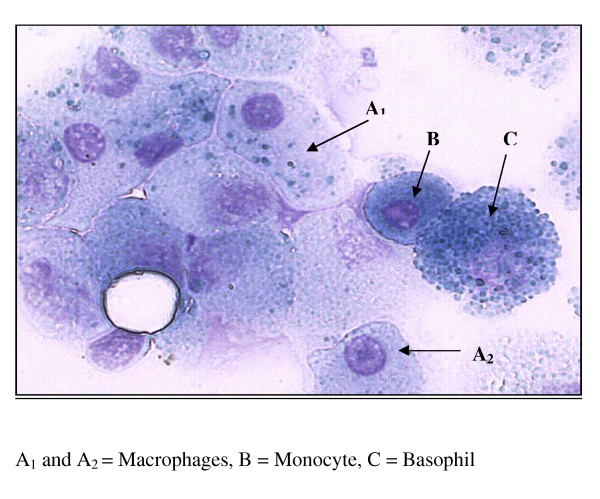
**BAL samples stained with Jenner/Giemsa stain to visualize cell morphology**.

Using the Jenner/Giemsa stain to visualize cell morphology, it was found that approximately 85% of cells obtained by bronchial lavage exhibited macrophage morphology (Figure 5). They displayed a large, reniform, purple nucleus and granular free, blue cytoplasm Fig 5). This percentage is in agreement with previously published data [17].

**Figure 6:**
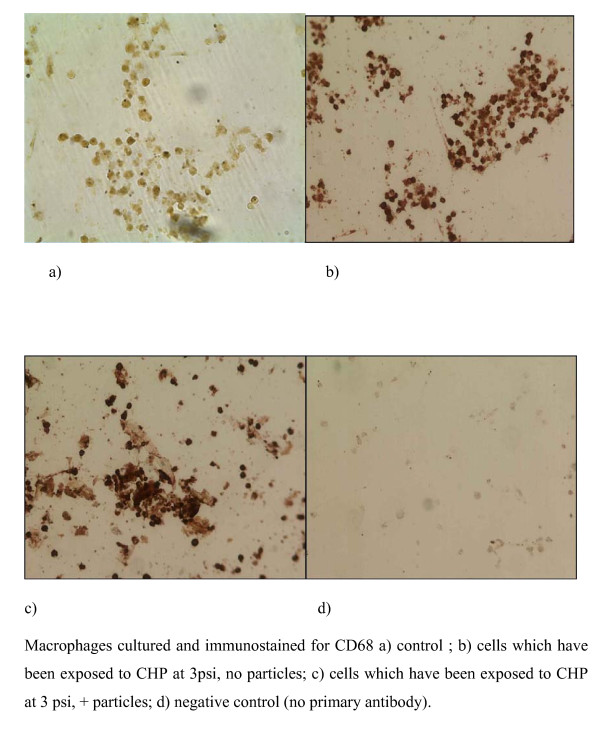
**Immunocytochemistry of BAL cell cultures using antibody to the macrophage marker CD68; images show a) control; b) cells which have been exposed to CHP at 3 psi, no particles; c) cells which have been exposed to CHP at 3 psi, + particles; d) negative control (no primary antibody)**.

**Figure 7:**
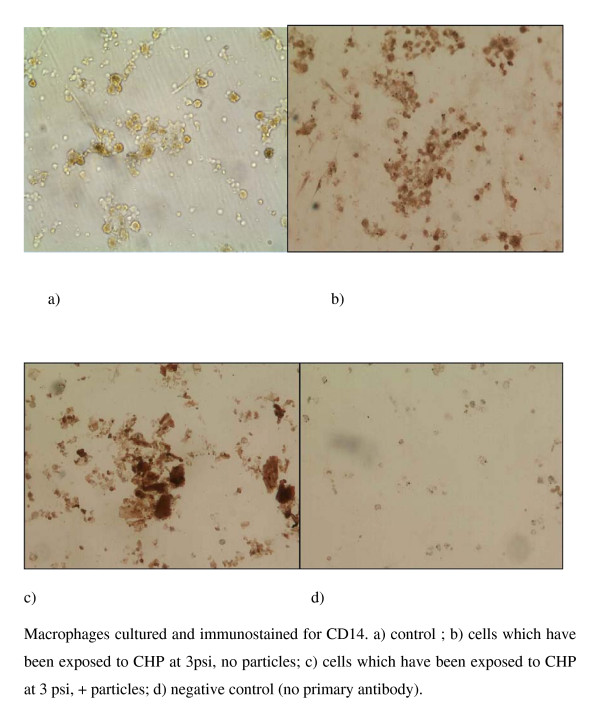
**Immunocytochemistry of BAL cell cultures using antibody to the macrophage marker CD14; images show a) control; b) cells which have been exposed to CHP at 3 psi, no particles; c) cells which have been exposed to CHP at 3 psi, + particles; d) negative control (no primary antibody)**.

Immunocytochemistry of the BAL cell cultures using antibodies to the macrophage cell surface markers CD68 and CD14, demonstrated that all the cells in the preparation stained positively for CD68 (Figure 6) and many stained positive for CD14 (Figure 7). It is of interest that there was an apparent increase in the depth of staining for both of these markers when cells were exposed to both CHP and particles (Fig 6, 7c), compared to exposure to CHP alone (Fig 6, 7b).

## Discussion

The main findings of this study were that alveolar macrophages from patients with a history of smoking responded to cotton particulate exposure by increasing the production of pro-inflammatory cytokines. CHP at 3 psi and 5 psi had no effect on cytokine production from these macrophages, but cells exposed to CHP at 5 psi and cotton particulates displayed increased cytokine production, most notably of IL-6. This suggests an interaction between CHP and particulate exposure in increasing alveolar macrophage cytokine production. This interaction was dependent on the pressure used, as it was only observed at the higher CHP. These observations were supported by experiments in THP-1 cells, where an interaction was observed for TNF production only at 5 psi and not 3 psi. CHP may be an important modulator of the response of alveolar macrophages to cotton particles, but the source of the cells may be a confounding factor which demands further investigation.

Particles of standard cotton dust were used in this study to investigate their effect, together with CHP, on alveolar macrophages. The problem of exposure of cotton workers to the particulates in organic dusts is well documented [18-20]. There is a large body of work which examines the effect of a variety of different particulates on lungs and lung cells but standardization of such particulates can be problematic and the variation in the materials could well be a confounding factor in the data obtained.

This study demonstrates the importance of particles of cotton dust and mechanical load, as CHP, to cytokine synthesis by lung macrophages from three different sources. It also highlights the different responses by these cells to the stimuli used, using the outcome measure of an increase in secretion of three pro-inflammatory cytokines, TNFα, IL-1β and IL-6.

It should be noted that *in vitro *alveolar macrophage studies are usually conducted at constant atmospheric pressure, which may not reflect the natural alveolar environment which is prone to pressure changes during ventilation. In order to study the effects of pressure changes on these cells, we used the same principle as in many previous studies [10-14]; cells were exposed to atmospheric pressure (14.69 psi), and a cyclical extra pressure (3 – 5 psi) was applied. Therefore, this model allows the hypothesis that small cyclical increments in pressure may have an effect on cytokine production to be tested. These pressures are higher than observed within normal humans lungs, as the alveolar pressure is lower than atmospheric pressure. However, lungs undergoing positive pressure ventilation are exposed to higher cyclical pressures, and our study shows that cyclical pressure may augment particle induced cytokine production. The effect of CHP was relatively small, although statistically significant.

Unlike our previous research [10-14], the effect of CHP on cytokine synthesis by the lung macrophages was small and did not attain statistical significance when compared to unpressurized cultures. This was found for all three types of alveolar macrophage tested.

However, exposure to cotton particles did cause statistically significant increases in cytokine synthesis in all three types of macrophage. In addition, there appears to be a trend in the data, whereby cultures exposed to both CHP and particles secrete more cytokine than cultures exposed to particles alone, but statistical significance was not found.

There was considerable variation in the amount by which secretion of an individual cytokine increased. In cultures of BAL macrophages, when we compared the levels of cytokines for cultures exposed to CHP and particles with control cultures, TNFα increased by nearly 500× and IL-6 by more than 300×, whereas the increase in IL-1β was very much lower (4×). The results from the macrophages isolated from biopsies were slightly different; whilst the greatest increase was still seen in TNFα (160×), IL-1β was increased more than IL-6 (16× compared to 2.5×).

It is of interest that, in cultures of BAL macrophages, when the ratios of the three cytokines are compared, a relationship of TNFα: IL-1β: IL-6 of approximately 1: 0.4: 0.5 is seen for endogenous synthesis (no stimulus) and also with exposure to pressure alone (data not shown). When cotton particles are included, this ratio is disturbed in a random fashion, suggesting disruption of a feedback mechanism. However, this ratio is not found with macrophages cultured from lung biopsies, demonstrating the differential response of the two cell types to CHP.

There has been extensive research (reviewed by Thorn)[21], on the response of macrophages from a variety of sources to cotton or dust particulates and the confounding effect of LPS, or the endotoxin often found on ambient samples of such materials [22-24]. These studies all show greater activation of macrophages when exposed to particulates and endotoxin. The cotton dust particles used here were selected to be of a size which macrophages can phagocytose (30–60% < 2 μm; 70–90% < 8 μm). In addition, they are coated with endotoxin, so these two properties together act to activate the macrophages in our study. In addition, these particles also mimic the in vivo contaminants to which people are exposed.

Whilst macrophages isolated from BAL had some sensitivity to mechanical load in the form of CHP, macrophages isolated from lung biopsies or from the cell line THP-1, appeared to exhibit comparatively less mechanosensitivity and their responses, whilst evident from the data, did not attain statistical significance. It is of interest that cells which did not show statistically significant mechanosensitivity in response to CHP, showed an increased response with the further addition of particles, which appeared to be dependent upon the level of CHP experienced by the cell. This result is not unexpected as we have shown previously, using peripheral blood macrophages, that secretion of cytokines exhibited an increased response to increased CHP [11]. However, the findings reported here suggest that CHP might sensitize macrophages to the effects of particulates. Whilst other workers have examined the effect of pressure on tissues ex vivo, such as pulmonary artery strips [7,25-27] ] this is, to the authors' knowledge, the first study of the combined effect of particulates and CHP on alveolar macrophages.

This research used only lung macrophages and does not attempt to examine the effect of CHP on other cells of the lung. However, it is likely that they may also be mechanosensitive and interaction between the cell types will be a confounding factor in any conclusions drawn from further studies. In addition, it is well established that there are complex interactions between the cytokines measured, but such activity is outwith the scope of this study.

The cell line THP-1 is commonly used in research into the lung as it has many of the characteristics of lung macrophages [28-31] and we wished to compare its response to CHP in relation to the primary lung macrophages we had used previously. In addition, as it is often difficult to obtain sufficient numbers of primary macrophages from patients for meaningful studies, we wished to see whether THP-1 cells would be a suitable alternative. However, the results show that, in our hands, THP-1 cells were less mechanosensitive than primary cells and their responses did not attain statistical significance. They were also less sensitive to particles of standard cotton dust than are primary cells.

The variation in responsiveness to particles between the different macrophages types leads one to speculate about the adapatability of macrophages to their environment. At the start of this study, we compared the response of BAL macrophages and peripheral blood macrophages to cotton dust exposure, demonstrating that peripheral blood macrophages rapidly underwent apoptosis on contact with the cotton dust particles, making any comparison impossible (data not shown). The apoptosis of peripheral blood macrophages and the apparent ability of alveolar macrophages to engulf cotton particles may indicate adaptation of a cell type to the different conditions found in different areas of the body. Other workers have shown that MP from three different sources showed different responses to particle exposure [32] but, to our knowledge, this is the first study to compare the response to CHP of MP from different sources. In addition, the data demonstrate that lung macrophages, which are continuously exposed to CHP, are much less sensitive to it than are blood macrophages, which are not normally exposed to this stimulus.

Finally, the data emphasise the differential sensitivity to both CHP and particulates of macrophages from different individuals. At present it is unclear why such variability exists and we aim to investigate the role of CD antigens in this complex response.

## Conclusion

This study shows that alveolar macrophages from patients with a history of smoking increased their production of pro-inflammatory cytokines in response to exposure to cotton particulates. It also demonstrated that CHP alone had little effect on cytokine production, but that CHP caused a small increase in cotton particulate stimulation of cytokine production, most notably of IL-6. This suggests an interaction between CHP and particulate exposure in increasing alveolar macrophage cytokine production. The study also highlighted that alveolar macrophages from different sources responded differently and that even cells from the same source showed some variation between individuals.

## Competing interests

The authors declare that they have no competing interests.

## Authors' contributions

SL performed all the laboratory techniques and experiments, prepared, analysed and helped to interpret the data and helped to prepare the manuscript. She also participated in the design of the study. DS helped with the design of the study, supplied the patient samples and also participated in the preparation of the manuscript. CEE conceived and designed the study, co-ordinated it, interpreted the data and drafted the manuscript. All authors read and approved the final manuscript.
